# Use of hyaluronic acid filler for enhancement of nipple projection following breast reconstruction: An easy and effective technique

**DOI:** 10.1016/j.jpra.2019.10.003

**Published:** 2019-11-05

**Authors:** Gloria R. Sue, Jennifer G. Seither, Dung H. Nguyen

**Affiliations:** Division of Plastic and Reconstructive Surgery, Department of Surgery, Stanford Health Care, 770 Welch Road, Suite 400, Stanford, CA 94304, USA

**Keywords:** Breast reconstruction, Filler injection, Hyaluronic acid filler, Nipple reconstruction

## Abstract

**Background:**

Breast reconstruction improves the psychological well-being of patients with breast cancer. Patients who complete nipple-areolar reconstruction are even more satisfied with their final reconstructive result. Nipple flattening is a common complication. We hypothesized that injectable soft-tissue filler can be used to augment nipple projection in patients who underwent breast reconstruction.

**Methods:**

This is a retrospective study of patients who underwent breast reconstruction and desired an enhanced postoperative nipple projection. The patients underwent a single session of injection with a hyaluronic acid filler as an outpatient. The filler was injected intradermally at the base of the nipple until the desired nipple projection was obtained.

**Results:**

Twelve patients and 22 breasts were included in this study. Enhanced nipple projection was observed in all cases, with an average increase of 3.0 mm in nipple height (range 2.5–4.5 mm). All injected nipples remained soft to the touch. All results were stable at a median of 7.5 months follow-up. No complications were observed.

**Conclusions:**

The use of injectable fillers for enhanced nipple projection is a useful adjunct treatment in patients undergoing breast reconstruction. Advantages include the ability to obtain nipple projection in patients who opt to forgo nipple-areola reconstruction with local flaps, to augment reconstructed nipples in patients with thin mastectomy skin flaps especially following implant-based reconstruction, and to improve projection of the native nipple following nipple-sparing mastectomy. Another benefit of this adjunct treatment is that the injection is reversible. Filler injection is a safe and simple solution to the problem of insufficient nipple projection.

## Introduction

Reconstruction of the nipple-areola complex is an integral component of breast reconstruction. Nipple-areola complex reconstruction is typically performed after the surgical creation of a breast mound, and is associated with an improved aestheticresult and increased patient satisfaction.^1^, ^2^, ^3^ This sense of reconstructive completeness is observed in both unilateral and bilateral nipple reconstructions.^4^ Nipple-areola complex reconstruction is thus a critical component of the breast reconstruction pathway that represents a restoration of a more natural appearance for the patient. Many surgical techniques have described for nipple-areola complex reconstruction. The most commonly utilized techniques include tattooing and the use of local flaps for nipple reconstruction.^5^, ^6^, ^7^, ^8^, ^9^ There is currently no gold standard technique for nipple-areola complex reconstruction.

Tattooing and local flap reconstructions each have their pros and cons. Tattooing is a nonsurgical reconstruction technique and can be readily performed in the outpatient setting. Recently, three-dimensional nipple-areola complex tattooing has been described by Halvorson et al., which utilizes artistic principles of light and shadow to create the illusion of nipple projection on a two-dimensional breast mound surface.^10^ However, there is a learning curve associated with tattooing and final aesthetic results may be operator-dependent. Additional complications of tattooing include prolonged healing time, scarring, and poor pigment retention.

Local flap reconstruction of the nipple is a simple surgical procedure that can be performed in the outpatient setting and provides nipple projection beyond the breast mound. Unfortunately, a common complication following local flap reconstruction of the nipple is the loss of nipple projection.^11^ Jabor et al. reported that among women who underwent nipple-areola complex reconstruction, patients were least satisfied with the projection of the reconstructed nipple, compared to other aesthetic outcomes such as color match, shape, size, texture, and position.^3^

Many techniques have been described to address the problem of nipple flattening following reconstruction. These reported projection improving techniques include the use of cartilage grafts (both rib and auricular), fat grafts, toe pulp grafts, and tissue-engineered grafts.^12^, ^13^, ^14^, ^15^, ^16^ A handful of case series have also reported on the use of injectable fillers for achieving improved nipple projection.^17^ These reports utilized calcium hydroxylapatite fillers,^18^ hydroxyethylmethacrylate and ethyl methacrylate fillers,^19^ and a filler composed of polymethylmethacrylate microspheres.^20^

Hyaluronic acid filler is a popular nonsurgical injectable used in facial rejuvenation to improve facial fullness.^21^ Compared to surgical facial rejuvenation techniques, filler injections are more convenient, less costly, and are associated with significantly less downtime. There are many hyaluronic acid filler products available, with varying levels of hyaluronic acid concentration, degree of cross-linking, particle size, and elastic modulus, which have implications for the indication of an injection.

The use of hyaluronic acid filler for enhancement of nipple projection has not been well characterized. Here, we report on the use of hyaluronic acid fillers for nipple projection in a series of patients.

## Patients and methods

We performed a retrospective study on patients undergoing breast reconstruction with the senior author (D.H.N.), from 2016 to 2017, who desired enhanced nipple projection following nipple sparing mastectomy or after nipple reconstruction with a local flap or 3D tattoo. The intervention consisted of injection of the hyaluronic acid filler, Juvederm Ultra XC (Allergan, Dublin, Ireland), at the base of the nipple. Juvederm Ultra XC was selected as the hyaluronic acid filler for this study because of its relatively short half-life and its reversibility with hyaluronidase. These injections were all performed in a clinical setting by the senior author. The injection site was prepped and draped in the usual sterile fashion prior to the procedure. The filler was then injected intradermally at the base of the nipple until the desired nipple projection was obtained, as determined by the patient.

Patient follow-up was performed for at least 6 months after the filler injection to assess the nipple projection and administer additional filler injection, if necessary. At follow-up, the nipples were assessed for the degree of projection and evaluated on its softness. Nipple projection was assessed using a ruler to measure the maximal projection of the nipple beyond the plane of the surrounding areola. All measurements were made by the senior author in this standard fashion.

## Results

A total of 12 patients were included in this study (Table 1). Of the 12 patients, 22 breasts underwent mastectomy for cancer treatment or cancer prophylaxis (Table 2). Thirteen of the breasts underwent nipple reconstruction, with 8 (61.5%) breasts undergoing reconstruction using local flaps, and 5 (38.5%) breasts undergoing tattooing.

**Table 1:** Demographics of patient cohort.

Characteristics	*N* = =12 (%)
Age (mean, in years)	51.3 (range 35–71)
Body mass index (mean, in kg/m^2^)	26.7 (range 24.1–33.2)
Race	
White	5 (41.7%)
Asian	1 (8.3%)
African American	4 (33.3%)
Hispanic	2 (16.7%)
History of diabetes	0 (0.0%)
History of radiation to the breast	3 (25.0%)
History of smoking	1 (8.3%)

**Table 2:** Surgical characteristics of patient cohort.

Surgical Characteristics	Number (%)
Number of patients (*N* = =12)	
Unilateral	2 (16.7%)
Bilateral	10 (83.3%)
Surgical indication per breast (*N* = =22)	
Cancer	16 (72.7%)
Cancer prophylaxis	6 (27.3%)
Type of mastectomy (*N* = =22)	
Simple mastectomy	13 (59.1%)
Nipple-sparing mastectomy	9 (40.9%)
Type of nipple reconstruction (*N* = =13)	
Flap	8 (61.5%)
Tattoo	5 (38.5%)

All patients underwent a single injection of Juvederm Ultra XC during the study period (Table 3). These injections were given 3–6 months after complete surgical healing following reconstruction. The average volume of filler used per nipple was 0.35 mL (range 0.25–0.6 mL) with a resultant average increase in nipple height of 3.0 mm (range 2.5–4.5 mm).

**Table 3:** Treatment summary of patient cohort.

Volume of filler (mean, range)	0.35 mL (range 0.25–0.6)
Height of nipple projection (mean, range)	3.0 mm (range 2.5–4.5)
Duration of follow-up (months)	7.5 months (range 6 to 9.6)
Complications	none

**Figure 1:**
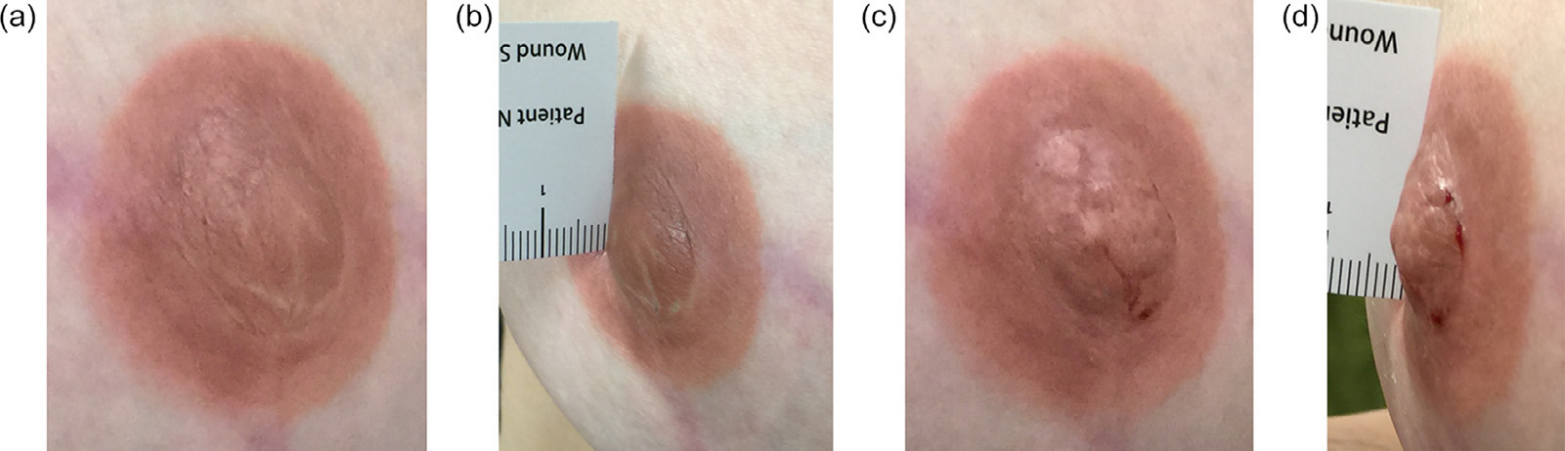
Patient who underwent bilateral simple mastectomies followed by implant-based breast reconstruction and nipple reconstruction using local flaps and tattooing, complicated by loss of projection in the nipples. (a) Prior to hyaluronic acid filler injection, anterior view. (b) Prior to hyaluronic acid filler injection, oblique view. (c) Immediate esthetic outcome following filler injection (0.6 mL) to nipple, anterior view. (d) Immediate esthetic outcome following filler injection (0.6 mL) to nipple, oblique view.

**Figure 2:**
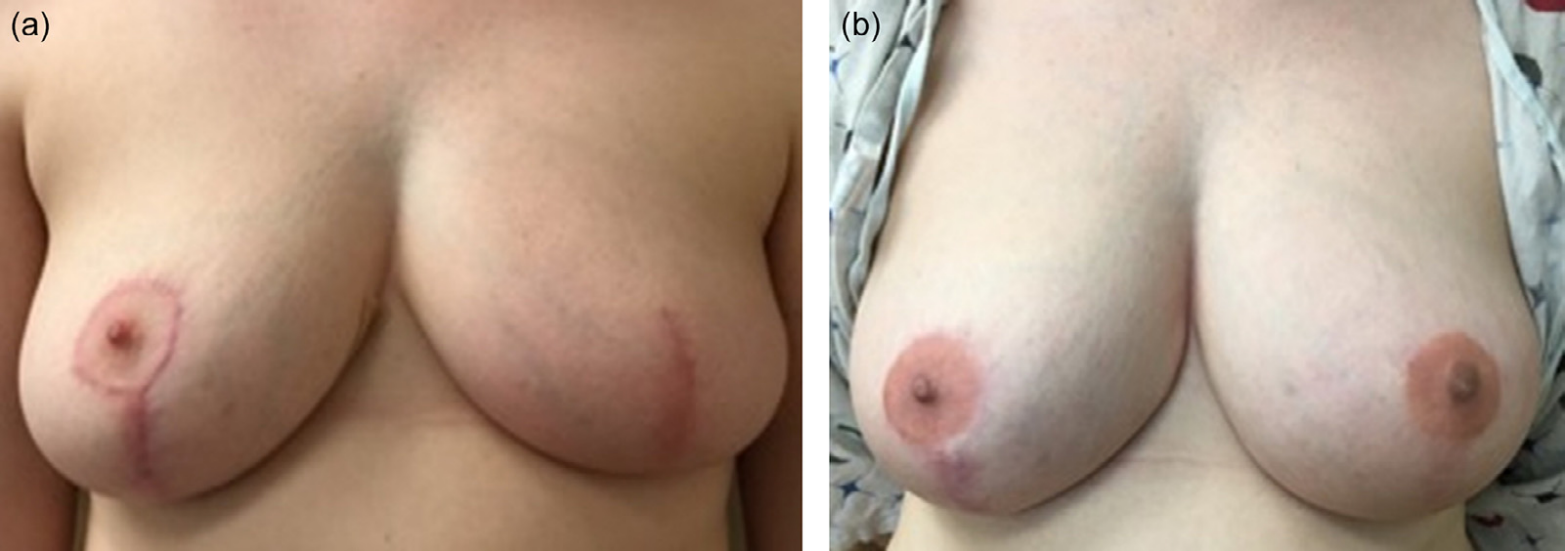
Patient who underwent left skin-sparing mastectomy with autologous breast reconstruction and nipple reconstruction with filler and 3D tattoo, with a right mastopexy for symmetry. (a) Preoperative photo. (b) 6 months after filler injection (0.3 mL) and 3D nipple tattooing to the left breast, with resultant good symmetry.

**Figure 3:**
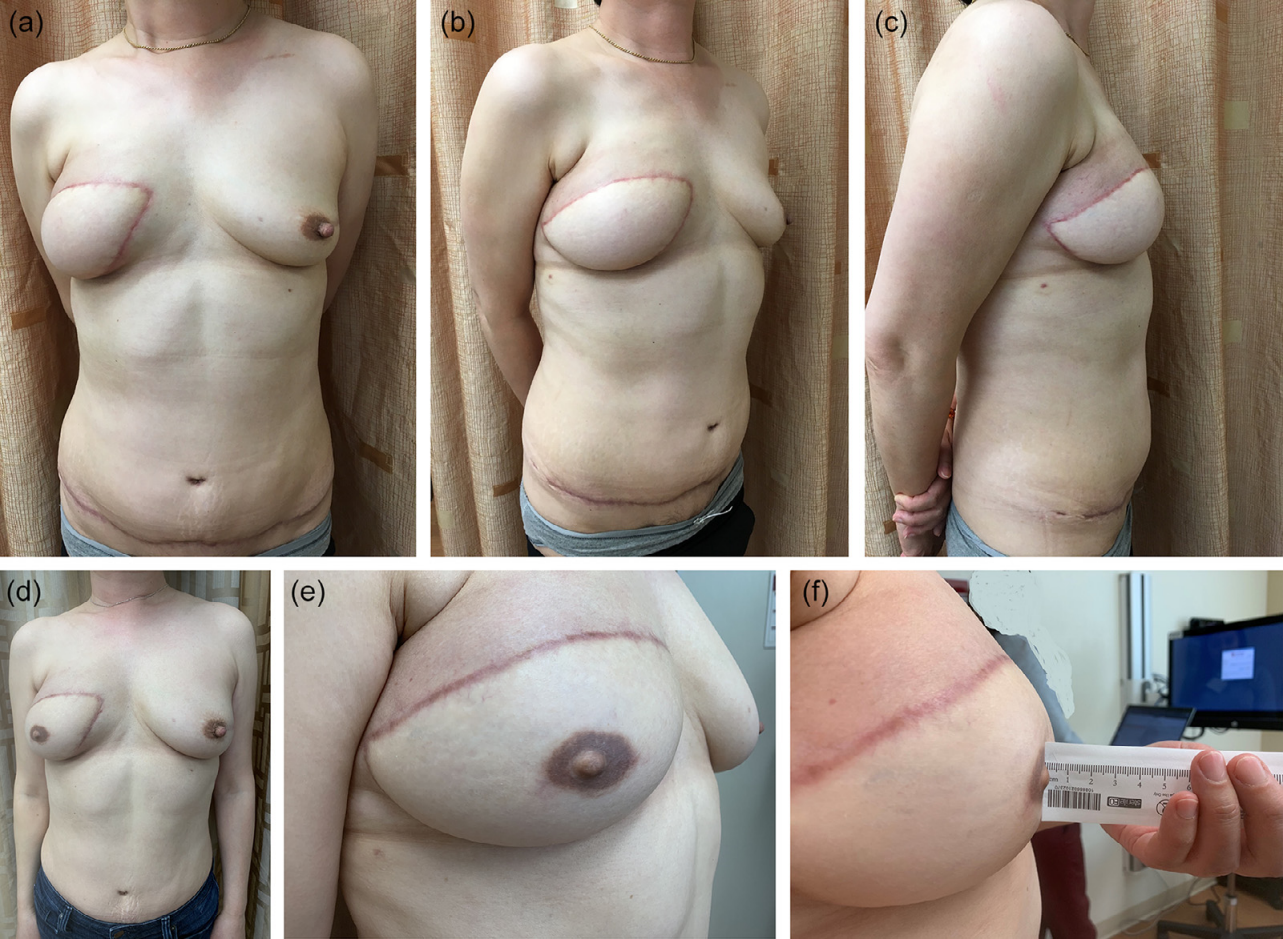
Patient who underwent right simple mastectomy with autologous breast reconstruction with (a) anterior view, (b) oblique view, and (c) lateral view. Same patient 7.5 months following right nipple reconstruction with filler and 3D tattoo with (d) anterior view, (e) oblique view, and (f) lateral view.

The patients were reevaluated at a mean of 7.5 months following filler injection (range 6 to 9.6 months). The nipple projection was unchanged compared to the postinjection nipple height for all patients at follow-up. Representative patients who underwent filler injection to the nipple are demonstrated in Figures. 1–3.

There were no complications associated with the filler injections to the nipple. All nipples remained subjectively soft to the touch following filler injection. All patients reported satisfaction with the cosmetic results.

## Discussion

This is a pilot study that demonstrates the safe and efficacious use of hyaluronic acid filler to achieve stable nipple projection in women who underwent breast reconstruction. All patients in this study had satisfactory increases in nipple projection following the hyaluronic acid filler injection. All patients were satisfied with their results at 6 months following the filler injection.

Hyaluronic acid is a glycosaminoglycan biopolymer naturally produced by the human body. Hyaluronic acid fillers, therefore, have a good safety profile as an injectable.^22^, ^23^, ^24^ Hyaluronic acid products were initially developed as an alternative to collagen. One benefit of hyaluronic acid compared to collagen is a longer duration of action.^24^ Hyaluronic acid is typically cross-linked to varying degrees to improve stability and longevity following injection. Another unique characteristic of hyaluronic acid fillers is that the degradation of the product is isovolemic, such that as the product degrades, the remaining hyaluronic acid binds additional water to maintain a constant overall volume.

Fillers that have been reported for use in nipple reconstruction include hydroxylapatite fillers,^18^ ethyl methacrylate fillers,^19^ and polymethylmethacrylate fillers.^20^ Unlike hyaluronic acid fillers, these are semipermanent (hydroxylapatite) and permanent (ethyl methacrylate and polymethylmethacrylate) fillers. Hydroxylapatite fillers are highly viscous but are associated with a predisposition for nodule formation, which creates an unsightly external contour.^25^ The effects of hydroxylapatite range from 9 months up to 5 years.^26^ In contrast, polymethylmethacrylate fillers are permanent. The permanence of this filler is secondary to the presence of nonbiodegradable microspheres.^27^^,^^28^ One downside of using this product is that patients must undergo skin testing at least 1 month prior to the injection, as sensitivity to the bovine collagen in these fillers must be excluded prior to the use.^29^

Fillers have also been reported for use in other aspects of breast surgery. Polyacrylamide hydrogel has been used for injectable filler for augmentation mammoplasty, breast tissue atrophy, and breast reconstruction.^30^ Polyacrylamide hydrogel is an atoxic, stable, and permanent injectable filler. It has been used for augmentation mammoplasty in Russia and China.^31^^,^^32^ One reported complication with this technique is that with large volume injections, the lactiferous ducts can become plugged. Given the nondegradable nature of the filler, once the ducts are clogged, local inflammation occurs often resulting in local infection and/or abscess.^32^ In women of breastfeeding age, this can result in loss of the ability to breastfeed.

Although we did not observe any complications in our patient cohort, it is possible that there are theoretical risks associated with filler injection to the base of the nipple. This potentially includes nipple necrosis from the pressure on the overlying skin secondary to the volume of injection. Careful discretion must be exercised with the injection with care not to over-inject the filler. Blanching of the overlying skin with injection is a sign that over-injection has occurred and the injection should cease at that point. Another possible complication is inadvertent injection of filler material into milk ducts. This can potentially cause duct obstruction with subsequent inflammation and infection, though this complication may be mitigated by the use of degradable fillers, where the occlusion could theoretically decrease over time.

The loss of projection observed in patients undergoing nipple reconstruction with local flaps is not well understood. Possible explanations include the absence of underlying connective tissue support for the local flaps and presence of wound contractile forces at the base of the reconstructed nipple.^18^ The injection of filler material to the dermis of the nipple directly augments the nipple and minimizes the counteracting effects of the above-listed factors. While hyaluronic acid filler is generally not considered a permanent solution for restoring volume, especially for its more common use in the face, we did not observe any loss of nipple projection among our cohort during the study period. We postulate that perhaps degradation of the filler is slower in the nipple compared to the comparatively more well-vascularized face. Although repeat filler injections in the future are expected, the patients have been satisfied with their results and have not required a repeat injection after 6–9 months.

Ultimately, the use of hyaluronic acid filler is a simple and effective method of improving nipple projection after nipple sparing mastectomy and after nipple reconstruction using flap or 3D tattoo. Its result can last at least 6 months with a single injection. In our pilot series of patients, there were no complications following this procedure. We were limited in our assessment of long-term results of a single hyaluronic acid filler injection by our duration of follow-up. The degradable nature of this type of filler mitigates the risk of potential complications observed in other similar studies, but potentially also limits the duration of response of therapy. Additional studies are needed to characterize the duration of effect of hyaluronic acid fillers to the nipple.

## Declaration of Competing Interest

None.
